# Effect of Adropin on Pancreas Exocrine Function in a Rat Model: A Preliminary Study

**DOI:** 10.3390/ani12192547

**Published:** 2022-09-23

**Authors:** Małgorzata Kapica, Iwona Puzio, Beata Abramowicz, Barbara Badzian, Siemowit Muszyński, Ewa Tomaszewska

**Affiliations:** 1 Department of Animal Physiology, Faculty of Veterinary Medicine, University of Life Sciences in Lublin, 20-950 Lublin, Poland; 2 Department and Clinic of Animal Internal Diseases, Faculty of Veterinary Medicine, University of Life Sciences in Lublin, 20-612 Lublin, Poland; 3 Department of Biophysics, Faculty of Environmental Biology, University of Life Sciences in Lublin, 20-950 Lublin, Poland

**Keywords:** adropin, trypsin, pancreatic secretion, vagal stimulation, rat

## Abstract

**Simple Summary:**

Adropin plays an important role in the regulation of energy homeostasis and metabolism. It enhances glucose tolerance, improves insulin resistance, ameliorates lipid metabolism, and has antihyperlipidemic activity. It has previously been detected in various organs, including the pancreas. Our aim was to investigate whether adropin affects pancreas exocrine secretion. The influence of different doses of adropin was tested in basal and stimulated conditions (CCK-8 and vagal stimulation). In addition, the effect of adropin has been studied under vagotomy and capsaicin deafferentation conditions. Our results indicate that adropin inhibits pancreas exocrine secretion both in the basal and stimulated conditions, whereas vagotomy and deafferentation abolish the pancreas responses to adropin. Based on the obtained results, it can be assumed that the effect of adropin on the pancreas is related to an indirect vagal mechanism. Nevertheless, this hypothesis requires further verification.

**Abstract:**

The aim was to investigate the potential effect of adropin (ADR) on pancreatic–biliary juice (PBJ) secretion (volume, protein content, trypsin activity) in a rat model. The animals were divided into control and five experimental groups: adropin, CCK-8 (CCK-8 stimulation), capsaicin (capsaicin deactivation of afferents), vagotomy (vagotomy procedure), and vagal stimulation (vagal nerve stimulation). The experiment consisted of four phases, during which vehicle (0.9% NaCl) and three ADR boluses (5, 10, and 20 µg/kg BW) were administered i.v. every 30 min. PBJ samples were collected from each rat at 15 min intervals after boluses. Exogenous ADR failed to affect the pancreatic responses after vagotomy and the capsaicin pretreatment and reduced the PBJ volume, protein outputs, and trypsin activity in the adropin, CCK-8, and vagal stimulation groups in a dose-dependent manner. In all these groups, volume of PBJ was reduced only by the highest dose of ADR (*p* < 0.001 for adropin group and *p* < 0.01 for CCK-8 and vagal stimulation groups), and the protein outputs were reduced by the administration of ADR 10 µg/kg BW (adropin and CCK-8 groups, *p* < 0.01 in both cases) and 20 µg/kg BW (*p* < 0.001 for adropin and CCK-8 groups, *p* < 0.01 for vagal stimulation group). The 10 µg/kg BW dose of ADR reduced the trypsin output in the CCK-8 group (*p* < 0.01), and the highest ADR dose reduced the trypsin output in the CCK-8 (*p* < 0.001) and vagal stimulation (*p* < 0.01) groups. In conclusion, adropin in the analyzed doses exhibits the negative feedback pathway. This mechanism seems to participate in the regulation of pancreatic juice secretion via an indirect vagal mechanism.

## 1. Introduction

In 2008, Kumar et al. [1] described a new peptide hormone, adropin (ADR), involved in the regulation of glucose and lipid homeostasis. The precursor of ADR is encoded by the energy homeostasis-associated gene (*ENHO*) expressed mostly in the brain and liver [1]. ADR34-76, which is derived from its 76-amino acid precursor, has biological activity. Over several years, the presence of adropin in blood and various organs, including the brain, kidneys, heart, blood vessels, pancreas, and small intestine, has been demonstrated [2,3,4,5,6].

**Figure 1:**
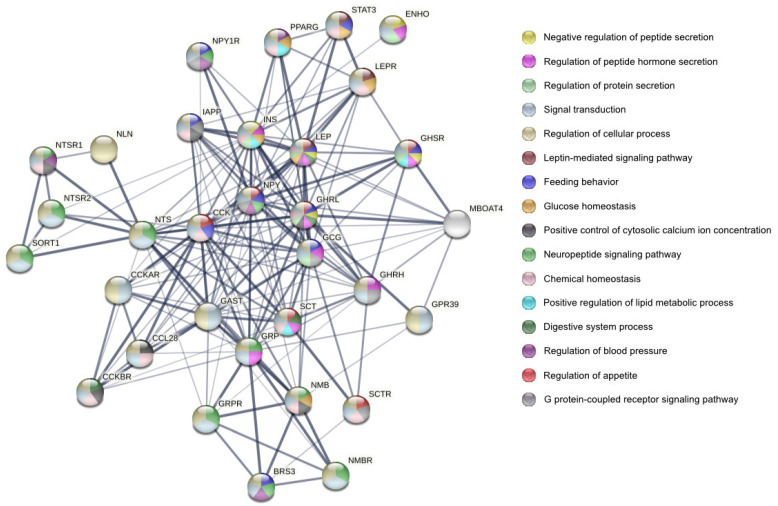
STRING protein–protein interaction diagram in various biological processes involving adropin (ENHO) and many other hormones and growth factors. The thickest edge indicates the highest confidence in protein–protein association. Network contains 32 nodes and 171 edges. The analysis of biological effects revealed that the proteins presented here are involved in many biological functions within the organism; https://string-db.org/, accessed on 25 August 2022. The proteins are described in Supplementary Materials.

ADR plays an important role in the regulation of energy homeostasis and metabolism. It enhances glucose tolerance, improves insulin resistance, and promotes the utilization of carbohydrates over fat in muscles [7,8]. Exogenous ADR has been reported to increase hepatic insulin sensitivity and suppress liver glucose production [9]. Moreover, ADR ameliorates lipid metabolism and has antihyperlipidemic activity [10,11]. Elevated ADR levels in blood can protect against such diet-induced disorders as obesity, glucose intolerance, insulin resistance, and fatty liver disorders [12]. Research results indicate that ADR participates in the pathogenesis of various cardiovascular disorders. Its low serum level is related to cardiac syndrome X, acute myocardial infarction, coronary atherosclerosis, and hypertension [13,14,15,16]. Moreover, ADR stimulates vascularization, blood flow, and vasodilatation [2,17]. The possible ADR interaction with other proteins and the biological process involved in this interaction is presented in Figure 1.

There is little information in the available databases about the relationship between adropin and gastrointestinal tract function. It is limited to a few reports. The gastroprotective potential of ADR was demonstrated in rats with indomethacin-induced gastric ulcers [18]. On the one hand, the ADR treatment reduced the activity of the gastric proton pump and the malondialdehyde level, but on the other hand, it upregulated superoxide dismutase and catalase activities. Moreover, ADR increased the content of gastric mucosal mucin, gastric PGE2, and VEGF cytoplasmic immunoreaction in the gastric mucosa and promoted Vegfr-2 expression [18].

The presence of ADR in the digestive tract was detected in the duodenum and jejunum. It was located in crypts, enterocytes, submucosa, muscularis externa, Brunner’s gland, and myenteric and submucosal plexus nerve [5]. Aydin et al. [3] indicated the presence of ADR in the acini of pancreas in rats. In the study mentioned above, ADR immunoreactivity was increased in animals with streptozotocin-induced diabetes. In turn, Billert et al. indicated a lack of *ENHO* mRNA expression in rat pancreatic islets and very low expression in INS-1E cells [19]. These data show that ADR is rather not produced by the endocrine pancreas of rats. However, ADR decreased insulin expression and secretion in INS-1E cells. INS-1E cells and pancreatic islets were characterized by mRNA expression of Gpr19, i.e., a putative ADR receptor [19].

Taking into account the very limited information on the role of adropin in the physiology of the gastrointestinal tract, the aim of our research was to investigate the potential effect of ADR on pancreatic juice secretion in a rat model in different conditions.

## 2. Materials and Methods

### 2.1. Animal Procedures

All experimental procedures performed in this study were approved by the Local Ethical Committee for Animal Experiments (Permit No. 54/2014, released on 9 April 2014; and Permit No. 61/2015, released on 30 June 2015).

### 2.2. Experimental Design

In vivo studies were performed on 36 male Wistar rats weighing 200–220 g. The rats were housed in cages in standard conditions at a controlled room temperature of 22 °C (±10%) and humidity of 55% (±10%) with a normal circadian rhythm: a 12 h day/night cycle. During the one-week period of acclimation to the new environment, food (commercial pellet chow—LSM Standard Rat and Mouse Chow) and water were available ad libitum. After the acclimatization period, the rats were randomly divided into six groups, one control and five experimental, each containing *n* = 6 animals:

1. Control (four 0.9% NaCl boluses);
2. Adropin (adropin boluses);
3. CCK-8 stimulation (CCK-8 stimulation and adropin boluses);
4. Capsaicin (capsaicin pretreatment and adropin boluses);
5. Vagotomy (vagotomy and adropin boluses);
6. Vagal Stimulation (vagal nerve stimulation and adropin boluses).

The night before the in vivo experiment, the animals were fasted, but water was not limited. All experiments were carried out at the same time in the morning. Surgical operations were performed under general anesthesia with an intramuscular (i.m.) injection of ketamine + xylazine (15 mg/kg BW + 35 mg/kg BW; Bioketan, Vetoquinol Biowet, Gorzów Wielkopolski, Poland + (Rometar, Bioveta a.s., Ivanovice na Hané, Czech Republic). For intravenous (i.v.) infusion, the right external jugular vein was prepared, and inserted tubes (silicone) were secured with ligatures. Continuous i.v. infusion of 0.9% NaCl (peristaltic pump speed: 2 mL/h) started immediately after cannulation and was maintained until the end of the experiment, except the i.v. application of adropin boluses. The bile and pancreatic juice mixture (PBJ) was collected from tubing (polyethylene) inserted in the common pancreatic–biliary duct after midline laparotomy. For the reintroduction of PBJ, second tubing was inserted into the duodenum through the pylorus. The anesthesia was maintained during the experiment by intraperitoneal administration of anesthetics, and rats’ body temperature was continuously monitored and maintained by heating lamps. The involvement of vagal nerves was examined following subdiaphragmatic vagotomy and capsaicin deafferentation, as described previously [20,21]. The Neuropack (Nihon Kohden Co., Tokyo, Japan) apparatus was used to stimulate the vagus nerve (rate, 1 Hz; duration, 0.2 ms; V-range, 2 mV; stim., 4.2 mA) [22,23].

### 2.3. In Vivo Experimental Protocol

**Figure 2:**
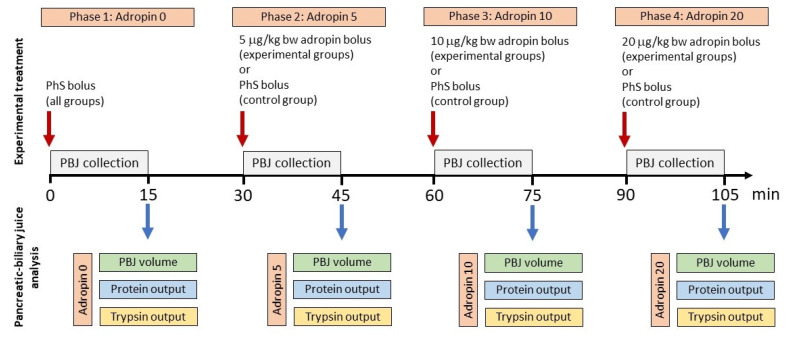
Scheme showing the experimental design.

Collection of PBJ started immediately after the surgery. PBJ was collected at 15 min intervals into 1.5 mL polyethylene tubes kept on ice. In four phases of the experiment vehicle (0.9% NaCl, Phase 1) and three adropin boluses (5, 10, and 20 µg/kg BW, Phase 2, 3, and 4, respectively) were administered i.v. every 30 min (Figure 2). The plasma half-life of ADR is still unknown; however, it is assumed that it is as short as other secretory proteins, i.e., several minutes [12]. Therefore, a 30 min interval was sufficient to suppress the effect of the previous dose.

Collected PBJ samples were used for further analysis. The volume of the PBJ samples was measured, and PBJ was aliquoted into 0.1 mL samples and stored in polypropylene tubes at −20 °C. The total protein content was estimated with the Lowry method in 96-well microwell plates according to the methods described previously [20]. Trypsin (EC 3.4.21.4) activity was estimated using the commercial Trypsin Activity Colorimetric Assay Kit K771-100 (BioVision Incorporated, Milpitas, CA, USA). The reading was made using a Benchmark Plus (Bio-Rad, Hercules, CA, USA) microplate spectrophotometer.

### 2.4. Statistical Analysis

The results are expressed as means ± SD. The statistical analysis was conducted using the statistical package GraphPad Prism 5.00 (GraphPad Software, San Diego, CA, USA). Statistical significance for all groups was investigated via one-way repeated-measures ANOVA, indicating significant differences. Mean differences between Adropin 0 (vehicle bolus, Phase 1) and the others were analyzed by Dunnett’s post-hoc test. Differences with *p* < 0.05 were considered significant.

## 3. Results

### PBJ Volume, Protein Outputs, and Trypsin Activity

**Figure 3:**
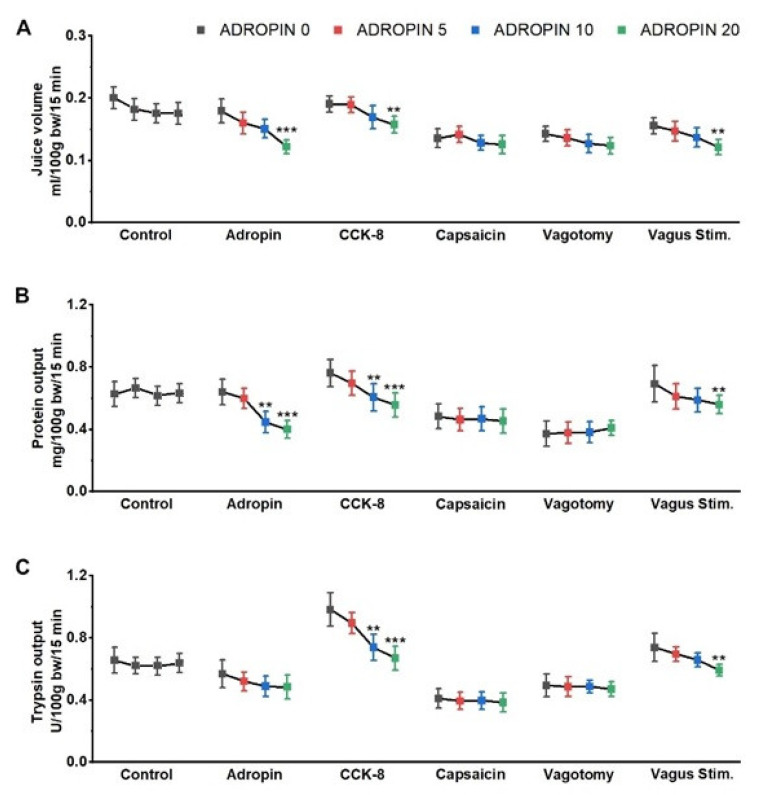
Effect of intravenous bolus infusions of adropin (5, 10, 20 µg/kg BW) on (**A**) the pancreaticobiliary juice (PBJ) volume, (**B**) protein output, and (**C**) trypsin output in control, basal (vehicle), CCK-8-stimulated (12 pmol /kg BW/ h), and basal secretion following vagotomy, capsaicin pretreatment in anesthetized rats. Each bar represents a 15 min PBJ sample after infusion of the vehicle alone or adropin. Each bar represents the mean ± SD (n = 6) of three independent experiments. Data were analyzed using one-way repeated measures ANOVA followed by Dunnet as post-test: ** *p* < 0.01, *** *p* < 0.001 vs. Adropin 0 (vehicle bolus, Phase 1).

The analysis of the obtained results indicated that the volume of PBJ in the basal conditions was significantly reduced by the highest dose of adropin (control vs. 20 µg/kg BW adropin: 0.179 and 0.122 mL/100g BW/15 min, respectively; *p* ˂ 0.01) (Figure 3A). Significant reduction was also observed in the CCK-8-stimulated animals (0.191 vs. 0.157 mL/100 g BW/15 min, *p* ˂ 0.001) and in the vagus-nerve-stimulated animals (0.156 vs. 0.121 mL/100 g BW/15 min, *p* ˂ 0.01). No significant changes in the PBJ volume were observed after adropin administration in rats after vagotomy and capsaicin deactivation of afferents (Figure 3A).

The protein outputs were reduced by the administration of 0, 5, 10, and 20 µg/kg BW ADR. The protein outputs were 0.64, 0.59, 0.44, and 0.39 mg/100g BW/15 min, respectively, in the basal conditions, 0.76, 0.69, 0.60, and 0.55 in the CCK stimulation group, and 0.69, 0.61, 0.58, and 0.56 in the vagus-nerve-stimulated animals. No responses to the application of adropin were observed in rats after vagotomy and deafferentation with capsaicin. The protein outputs at the doses of 0, 5, 10, and 20 µg/kg BW ADR were 0.37, 0.37, 0.38, 0.40 mg/100 g BW/15 min, respectively, in the vagotomized rats and 0.48, 0.46, 0.46, and 0.45 in the rats subjected to the capsaicin pretreatment (Figure 3B).

The effect of ADR on the trypsin output was dose related. The highest dose of ADR reduced the trypsin output significantly in CCK (0.98 vs. 0.67) and vagus-nerve-stimulated (0.74 vs. 0.59) rats. No statistical differences in trypsin outputs were observed after the vagotomy and deafferentation with capsaicin. The trypsin outputs at the ADR doses of 0, 5, 10, and 20 µg kg/BW were 0.49, 0.48, 0.48, 0.47 U/100g BW/15 min, respectively, in the vagotomized rats and 0.41, 0.39, 0.39, and 0.38 in the capsaicin pretreatment group (Figure 3C).

## 4. Discussion

The gastrointestinal tract (GI tract) and pancreas are known to provide hormonal signals to the central nervous system. These hormones include ghrelin, obestatin, gastric leptin, apelin, and nesfatin-1 secreted by the stomach and intestines and released in small amounts from the endocrine pancreas [24,25,26,27,28,29]. As shown previously, these hormones can regulate the secretion of pancreatic juice [20,21,30,31,32,33]. These signals send feedback to the region of the central nervous system that controls energy balance [34].

ADR, involved in regulating the body’s energy homeostasis, is secreted by many types of tissues in the human body [35]. First, after ADR was discovered, most researchers focused on its role in lipid and carbohydrate metabolism and insulin resistance [1,7,8,9,10,11]. Kumar et al. [1] showed that serum adropin levels were elevated proportionally to the increase in dietary fat intake and suppressed by fasting. Moreover, the immunopositive reaction for ADR in the duodenum, jejunum, crypts, and enterocytes located along the entire length of the villi suggests its role in the functioning of the GI tract [5]. In the pancreas, ADR was detected in acinar cells [3] and in the capillaries of the islets of Langerhans [36]. Aydin and colleagues [3] observed that the concentration of adropin in type 1 diabetic rats was increased in serum and many organs (pancreas, liver, kidney, muscles, and brain), while Polkowska et al. [37] reported that the serum level of adropin was decreased in children with type 1 diabetes. Moreover, in rats with streptozotocin-induced diabetes, the highest level of ADR was observed in the pancreas [3]. However, the data on ADR levels in organisms with type II diabetes are inconclusive. Both a decrease [15,38,39,40,41] and an increase [42] in the adropin level have been observed. In addition, a decrease in the insulin level depending on adropin overexpression or adropin treatment in experimental animals was observed [1]. Recent in vitro studies have confirmed that adropin is involved in the regulation of the activities of pancreatic beta cells, as evidenced by a reduction of insulin release [19].

Currently, there is no information in the scientific literature about the role of adropin in pancreatic exocrine functions. Our study shows for the first time that the administration of adropin influences pancreatic juice secretion, as we demonstrate that exogenous adropin can reduce the volume of pancreatic juice secretion and decrease protein outputs and trypsin activity in a dose-dependent manner. Adropin inhibited the exocrine function of the pancreas in both the basal and CCK-8-stimulated conditions. The strongest effect of adropin administration on the PBJ volume was observed after the application of the highest dose of ADR (20 µg/kg BW), while the most potent effect on the protein and trypsin outputs was noted after the administration of 10 and 20 µg/kg BW.

In the last two decades, our team studied the influence of newly discovered hormones on pancreatic juice secretion. In earlier studies, we found that the i.v. administration of exogenous ghrelin and apelin decreased [20,32], while obestatin stimulated pancreatic juice secretion [21,31]. The above-mentioned studies have also shown that the method of hormone administration plays an important role in its influence on the pancreas. In the case of apelin, we observed that the intravenous administration decreased pancreatic juice secretion while the intraduodenal administration increased the volume of PBJ [33].

Moreover, our previous studies showed that the i.v. administration of apelin-13 boluses decreased pancreatic blood flow [33]. In turn, the administration of increasing doses of ghrelin [43] or obestatin [44] did not affect pancreatic blood flow. In the circulatory system, Lovren et al. [2] showed that adropin was expressed in endothelial cells, regulated angiogenesis, and increased blood flow. In another study, Howarth et al. [45] reported that ADR improved cardiac function and coronary flow. Currently, there is no information on the effect of ADR on pancreatic blood flow.

The vagus nerve participates in the regulation of pancreatic juice secretion. Several studies have shown that electrical vagal stimulations induced the release of CCK-8 into the circulation [22,23,46]. Our study demonstrated that the vagal stimulation increased the volume and the protein and trypsin outputs in pancreatic juice. However, adropin administration diminished the values of the above-mentioned parameters during vagal stimulation. Moreover, in the present study, vagotomy, and deafferentation with capsaicin totally abolished pancreatic responses (protein and trypsin outputs), suggesting that adropin influences the exocrine pancreas depending on the maintenance of the integrity of sensory nerves. These results confirm our assumption that adropin acts via the duodenal CCK–vagal mechanism. In our previous studies on ghrelin [20], obestatin [21,31], leptin [30], and apelin [33], we also observed CCK-dependent regulation of pancreatic juice secretion. The results of the research provide new information about the hormonal regulation of the exocrine pancreas via the CCK–vagal mechanism. Unfortunately, our research does not fully explain the mechanism of the adropin action on the pancreas and PBJ secretion. This partially results from one of the limitations of this study, i.e., examination of the PBJ output of only one of the pancreatic enzymes, trypsin. Therefore, there is a need for further research in this field, including the analysis of the activity of other pancreatic enzymes such as lipase and amylase.

## 5. Conclusions

In conclusion, adropin administered in the analyzed doses exhibits a negative feedback pathway. This mechanism seems to participate in the regulation of pancreatic juice secretion via an indirect vagal mechanism. However, the current knowledge about adropin with its key role and mechanism in the regulation of many physiological and pathophysiological conditions, including pancreatic function, is insufficient, and this issue should be further investigated not only in the rat model but also in other non-human-model animals.

## Data Availability

The data presented in this study are available on request from the corresponding authors.

## Supplementary Materials

The following supporting information can be downloaded at: https://www.mdpi.com/article/10.3390/ani12192547/s1, The list protein abbreviations in STRING protein–protein interaction diagram in Figure 1.
