# Supplementary material for: Effect of Adropin on Pancreas Exocrine Function in a Rat Model: A Preliminary Study
Source: Animals (Basel). 2022 Sep 23;12(19):2547. doi: 10.3390/ani12192547 (PMC9558541; doi:10.3390/ani12192547)
Supplement: Supplementary file 1 [file animals-12-02547-s001.zip › animals-1910062-supplementary.pdf]

Effect of Adropin on Pancreas Exocrine Function in a Rat Model - A Preliminary Study

Małgorzata Kapica, Iwona Puzio, Beata Abramowicz, Barbara Badzian, Siemowit Muszyński, Ewa Tomaszewska

The list of protein abbreviations in STRING protein-protein interaction diagram in Figure1

BRS3	Bombesin receptor subtype-3;
CCK	Cholecystokinin;
CCKAR	Cholecystokinin receptor type A;
CCKBR	Gastrin/cholecystokinin type B receptor;
CCL28	C-C motif chemokine 28;
**ENHO**	**Adropin (Energy homeostasis associated);**
GAST	Gastrin;
GCG	Glucagon;
GHRH	Somatoliberin;
GHRL	Ghrelin/Appetite-regulating hormone;
GHSR	Growth hormone secretagogue receptor type 1;
GPR39	G-protein coupled receptor 39;
GRP	Gastrin-releasing peptide;
GRPR	Gastrin-releasing peptide receptor;
IAPP	Islet amyloid polypeptide;
INS	Insulin;
LEP	Leptin;
LEPR	Leptin receptor;
MBOAT4	Ghrelin O-acyltransferase;
NLN	Neurolysin;
NMB	Neuromedin-B;
NMBR	Neuromedin-B receptor;
NPY	Pro-neuropeptide Y;
NPY1R	Neuropeptide Y receptor type 1;
NTS	Neurotensin/neuromedin N;
NTSR1	Neurotensin receptor type 1;
NTSR2	Neurotensin receptor type 2;
PPARG	Peroxisome proliferator-activated receptor gamma;
SCT	Secretin;
SCTR	Secretin receptor;
SORT1	Sortilin;
STAT3	Signal transducer and activator of transcription 3;
